# Upregulation of COL6A1 is predictive of poor prognosis in clear cell renal cell carcinoma patients

**DOI:** 10.18632/oncotarget.4860

**Published:** 2015-08-07

**Authors:** Fangning Wan, Hongkai Wang, Yijun Shen, Hailiang Zhang, Guohai Shi, Yao Zhu, Bo Dai, Dingwei Ye

**Affiliations:** ^1^ Department of Urology, Fudan University Shanghai Cancer Center, Shanghai, People’s Republic of China; ^2^ Department of Oncology, Shanghai Medical College, Fudan University, Shanghai, People’s Republic of China

**Keywords:** COL6A1, clear cell renal cell carcinoma, prognosis, tumorigenesis

## Abstract

**Background**: The extracellular matrix (ECM) is reported to play an important role in tumorigenesis and progression. Collagen VI is an important ECM protein. In this study, we investigated the potential role of the COL6A1 gene, which encodes the α1 polypeptide of collagen VI, in the biological functions involved in the progression and outcome of clear cell renal cell carcinoma (ccRCC).

**Materials and methods**: A total of 288 ccRCC patients who underwent radical nephrectomy (RN) or nephron sparing nephrectomy (NSS) at Fudan University Shanghai Cancer Center (FUSCC) were enrolled. Total RNA was extracted from frozen samples obtained from the tissue bank of FUSCC and expression of COL6A1 was determined by qRT-PCR. The clinical relationship between COL6A1 expression and ccRCC prognosis was analyzed. These data were then validated in the Cancer Genome Atlas (TCGA) cohort. We also investigated the effect of COL6A1 overexpression in a xenografted tumor model in nude mice *in vivo*.

**Results**: In multivariate analysis of TCGA cohorts, COL6A1 high expression was predictive of poor prognosis in ccRCC patients’ overall survival (OS) (HR: 2.588 95%CI 1.616–4.146) and disease free survival(DFS) (HR: 3.106 95%CI 1.534–6.288). In FUSCC cohorts, after adjusted for relevant factors, the COL6A1 expression indicates poor prognosis in ccRCC patients’s OS (HR 2.211; 95% CI, 1.360–8.060) and DFS (HR 3.052; 95%CI, 1.500–6.210). COL6A1 overexpression promoted tumor growth in xenografted nude mice.

**Conclusion**: Increased COL6A1 expression correlates with poor prognosis in ccRCC patients. Moreover, COL6A1 stimulates tumor growth *in vivo*.

## INTRODUCTION

Renal cell carcinoma (RCC) accounts for approximately 2%–3% of all malignancies. In the United States, an estimated 64,770 Americans will be diagnosed with renal cancer and 13,570 will die of the disease in 2012 [1]. The most important prognostic factors of RCC include tumor size, histological subtype, nuclear grade, local extent of the tumor and evidence of metastatic disease at presentation [2]. Despite recent advances in diagnosis and treatment strategies, the prognosis for advanced and metastatic RCC patients remains poor. A clearer understanding of the molecular mechanisms of RCC and the identification of novel biomarkers are still required to clarify RCC etiology and susceptibility.

Collagen VI is a major extracellular matrix (ECM) protein, which forms a network of beaded microfilaments that interact with other ECM molecules and provide structural support for cells [3]. Studies have also indicated that collagen VI triggers signaling pathways that regulate cell apoptosis [4], inflammation [5] and even tumor progression [6]. Collagen VI is composed of three major polypeptide chains (α1, α2 and α3) encoded by distinct genes. The COL6A1 gene encodes the α1 chain and is usually involved in tumor metastasis. A report by Chiu indicated that COL6A1 knockdown suppresses the metastatic ability of lung cancer cells, whereas overexpression of COL6A1 has the opposite effect [7]. In a previous global secretome analysis, high expression of COL6A1 was found to promote *in vivo* bone metastasis [8]. To date, the majority of studies relating to the oncogenic functions of this gene have been mainly profiling studies, while the potential role of COL6A1 in RCC and its biological functions on the initiation, progression and outcome of the disease remain unknown.

## RESULTS

### Clinical factors in TCGA and FUSCC cohorts

A total of 497 patients from TCGA cohort and 288 patients from the FUSCC were enrolled in the current study. The two cohorts were similar in terms of age range and sex distribution. Detailed clinicopathological data are shown in Table 1. The expression levels of COL6A1 in the two cohorts were both nearly normal distributed (data not shown); therefore, we divided the two cohorts into low or high expression groups according to median expression level. There were 17 patients underwent neoadjuvant treatment in TCGA cohort. COL6A1 expression and clinicopathological factors were evaluated (Table 2). COL6A1 was not related to clinicopathological factors in both cohorts.

**Table 1:** Clinicopathologic characteristics of patients with ccRCC in the TCGA and FUSCC Cohorts

Variable	TCGA Cohort (*N* = 497)			FUSCC Cohort (*N* = 288)	
		*N*	%	*N*	%
Age (y)		61(26 to 90)		55(26 to 86)	
Sex	Male	327	65.80	198	68.75
	Female	170	34.20	90	31.25
pT	T1	245	49.20	201	69.79
	T2	65	13.10	39	13.54
	T3	176	35.40	38	13.19
	T4	11	2.20	10	3.47
pN	N0	233	46.88	273	94.79
	N1	17	3.42	6	2.08
	Nx	247	49.70	9	3.13
M	M0	419	84.31	275	95.49
	M1	78	15.69	11	3.82
	Mx	NA	NA	2	0.69
Fuhrman Grade	1 & 2	221	44.50	138	47.92
	3 & 4	269	54.10	150	52.08
	x	7	1.40	NA	NA
Pathological AJCC Stage	Stage I	240	48.29	198	68.75
	Stage II	54	10.87	36	12.50
	Stage III	124	24.95	38	13.19
	Stage IV	79	15.90	16	5.56
COL6A1	Low	248	49.90	144	50.00
	High	249	50.10	144	50.00

TCGA: The Cancer Genome Atlas; FUSCC: Fudan University Shanghai Cancer Center; NA: not applicable; AJCC: American Joint Committee on Cancer; pT : pathological *T* stage, pN: pathological *N* stage; M: M stage.

**Table 2:** Multivariate logistic regression analysis of clinicopathological factors and high COL6A1 expression level

Variables	High COL6A1* in TCGA cohorts		High COL6A1* in FUSCC cohorts	
	OR(95%CI)	*p*	OR(95%CI)	*p*
Age	0.996(0.974–10.19)	0.742	1.014(0.995–1.035)	0.155
Gender^#^	1.330(0.767–2.303)	0.310	0.809(0.480–1.364)	0.427
pT^$^	1.254(0.555–2.837)	0.586	2.339(0.570–9.594)	0.238
pN^#^	1.785(0.557–5.718)	0.329	2.874(0.263–31.379)	0.387
M^#^	1.321(0.389–4.481)	0.655	0.895(0.140–5.721)	0.906
Fuhrman Grade^#^	1.210(0.684–2.142)	0.512	0.777(0.467–1.294)	0.332
AJCC Stage^$^	0.996(0.413–2.260)	0.937	0.521(0.128–2.127)	0.364

* The cut-point of COL6A1 expression was defined as the median.

Abbreviations: 95% CI, 95% confidence interval; TCGA: The Cancer Genome Atlas; FUSCC: Fudan University Shanghai Cancer Center, AJCC American Joint Committee on Cancer

# Dichotomic variable: male patients versus female patients; pathological lymph node positive or otherwise; distant metastasis or otherwise; Fuhrman grade >2 or otherwise.

$ Categorical variable: Pathological *T* stage were defined as 1,2,3,4; AJCC stages were defined as I, II, III or IV.

### COL6A1 expression was an independent prognostic factor for OS and DFS in the TCGA cohort

**Figure 1:**
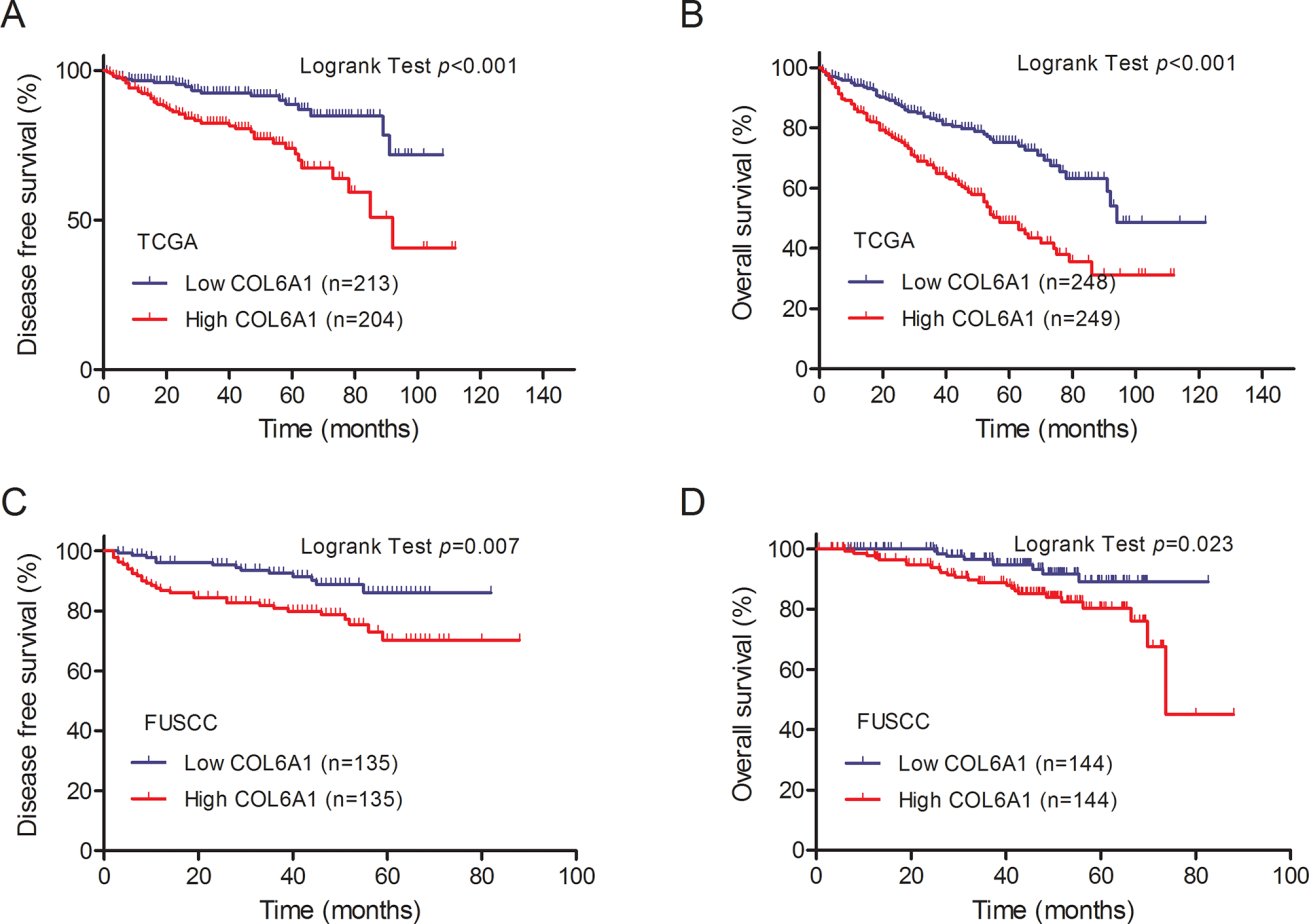
Kaplan–Meier plots of survival are shown according to COL6A1 expression **A.** Kaplan-Meier estimates of disease-free survival (DFS) in the Cancer Genome Atlas (TCGA) cohort. The median survival time of high and low expressers was 79.1 versus 95.3 months. **B.** Kaplan–Meier plots of overall survival (OS) in TCGA cohort. The median survival time of high and low expressers was 85.7 versus 108.6 months. **C, D.** Kaplan–Meier estimates of DFS and OS in FUSCC cohort. The median survival time of high and low expressers was 69.7 versus 75.6 months for DFS and 71.4 versus 78.1 for OS.

In the TCGA cohort, the Kaplan–Meier plot demonstrated that COL6A1 high expressers were associated with poor OS and DFS (Figure 1A, 1B). In univariate Cox proportion hazard ratio analysis, age, *T* stage, positive lymph nodes, metastasis, tumor stage and high COL6A1 expression were significantly associated with poor prognosis in terms of OS of patients with ccRCC in the TCGA cohorts (*P* < 0.001, Table 3). Multivariate analysis after adjustment for all the potential prognostic factors indicated that age and COL6A1 expression level (HR: 2.588 95%CI 1.616–4.146) were the only two predictors of OS (all *P* < 0.01, Table 3). In the TCGA cohort, high COL6A1 expression was an independent prognostic factor for DFS in 417 patients with localized ccRCC (HR: 3.106 95%CI 1.534–6.288, *P* < 0.01, Table 4) after adjustment for age, sex, *T* stage, *N* stage and AJCC stage.

**Table 3:** Univariate and multivariate Cox regression of Overall survival for patients with ccRCC in the TCGA cohorts

Variables	Univariate		Multivariate	
	HR(95%CI)	*p*	HR(95%CI)	*p*
Age	1.030 (1.017–1.043)	**0.000**	1.029 (1.009–1.049)	**0.004**
Gender				
female	1		1	
male	0.914 (0.663–1.259)	0.581	1.291 (0.803–20.76)	0.292
pT				
1	1		1	
2	1.486 (0.858–2.574)	0.158	0.339 (0.055–2.094)	0.244
3	3.524 (2.452–5.065)	**0.000**	0.575 (0.122–2.702)	0.483
4	11.674 (5.842–23.329)	**0.000**	0.932 (0.175–4.960)	0.934
pN				
0	1		1	
1	2.618 (1.390–4.929)	**0.003**	0.725 (0.273–1.923)	0.725
pM				
0	1		1	
1	4.429 (3.208–6.115)	**0.000**	2.180 (0.213–22.278)	0.511
Fuhrman Grade				
1 & 2	1		1	
3 & 4	2.706 (1.886–3.884)	**0.000**	1.664 (0.968–2.858)	0.065
Stage				
I	1		1	
II	1.175 (0.602–2.294)	0.635	2.449 (0.332–18.071)	0.380
III	2.850 (1.875–4.332)	**0.000**	3.786 (0.752–19.066)	0.106
IV	6.904 (4.616–10.326)	**0.000**	4.847 (0.305–76.971)	0.263
COL6A1				
low	1		1	
high	2.236 (1.613–3.100)	**0.000**	2.588 (1.616–4.146)	**0.000**

Abbreviations: 95% CI, 95% confidence interval; HR, hazards ratio; TCGA, The Cancer Genome Atlas.

* Bold type indicates statistical significance.

**Table 4:** Univariate and multivariate Cox proportional hazards analysis of disease-free survival for 417 patients with localized ccRCC

Variables	Univariate		Multivariate	
	HR (95%CI)	*P*	HR (95%CI)	*P*
Age	1.010 (0.990–1.030)	0.343	1.019 (0.991–1.048)	0.185
Sex		0.102		0.774
Female	1		1	
Male	1.591 (0.912–2.775)		1.115 (0.531–2.340)	
pT		**0.000**		0.943
1	1		1	
2	2.496 (1.192–5.224)	**0.015**	1.185 (0.090–15.690)	0.897
3	4.123 (2.355–7.220)	**0.000**	1.489 (0.127–17.435)	0.751
pN		**0.000**		0.100
0	1		1	
1	7.389 (3.016–18.101)		2.972 (0.811–10.884)	
Fuhrman Grade		**0.009**		0.780
1 & 2	1		1	
3 & 4	2.022 (1.196–3.418)		0.899 (0.426–1.897)	
Stage		**0.000**		0.531
I	1		1	
II	2.192 (0.989–4.858)	0.053	1.982 (0.124–31.721)	0.629
III	4.631 (2.645–8.110)	**0.000**	4.067 (0.317–52.183)	0.281
COL6A1		**0.001**		**0.002**
Low	1		1	
High	2.530 (1.497–4.275)		3.106 (1.534–6.288)	

Abbreviations: 95% CI, 95% confidence interval; HR, hazards ratio; TCGA, The Cancer Genome Atlas;

* Bold type indicates statistical significance.

### COL6A1 expression is associated with clinical outcome in the FUSCC cohort

**Figure 2:**
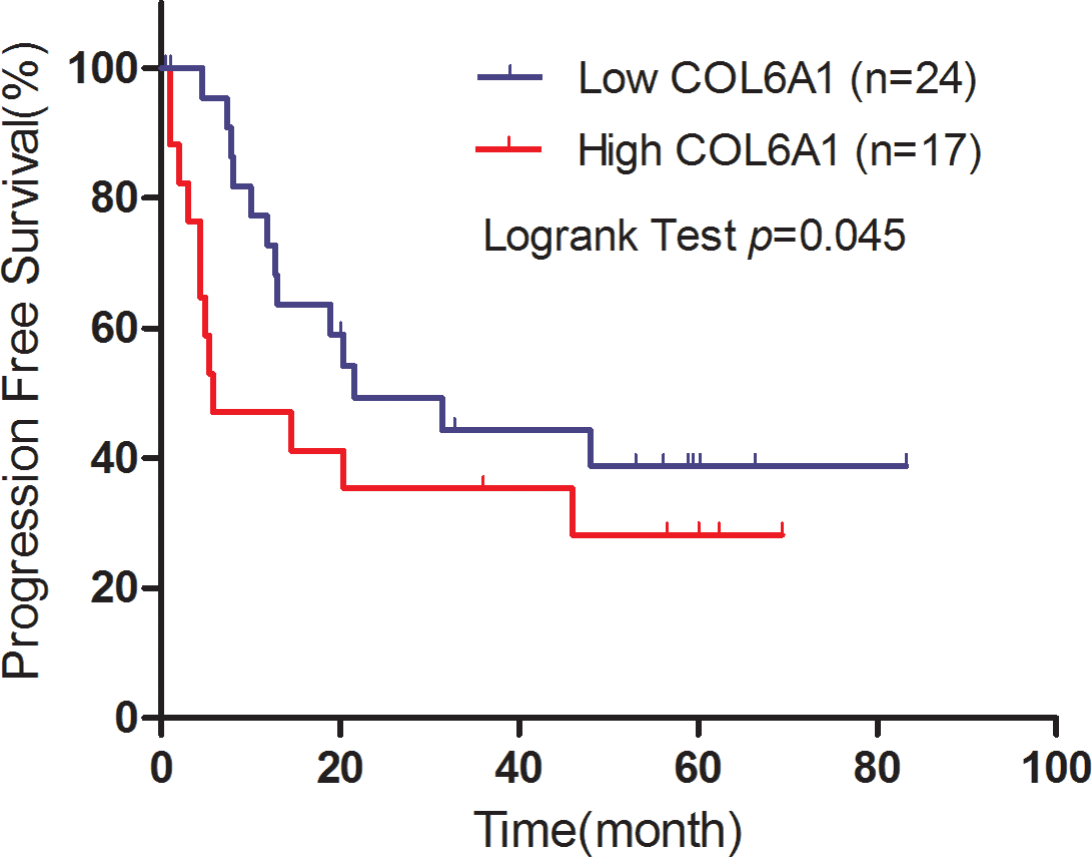
Kaplan–Meier plot of progression free survival (PFS) is shown according to COL6A1 expression A total of 41 patients received sunitinib treatment. The median survival time of high and low expressers was 5.8 versus 21.67 months.

In the FUSCC cohort, Kaplan–Meier analysis revealed that high COL6A1 expression was associated with poor DFS (*N* = 270, *P* = 0.007) and OS (*N* = 288, *P* = 0.023) (Figure 1C, 1D). Cox proportional hazards analysis showed similar results indicating that high COL6A1 expression is significantly associated with decreased DFS (HR 3.052; 95%CI, 1.500–6.210 [*P* = 0.002]) and OS (multivariate HR 2.211; 95% CI, 1.360–8.060 [*P* = 0.008])(Table 5). These data indicate that COL6A1 expression is associated with poor survival in patients with ccRCC. 41 patients accepted sunitinib target therapy in FUSCC cohort. In the subgroup analysis, high COL6A1 expression was associated with poor progression free survival (*P* = 0.045, Figure 2).

**Table 5:** Multivariate Cox proportional Hazards analysis of OS and DFS for patients with ccRCC in the FUSCC cohorts

Variables	DFS		OS	
	HR (95%CI)	*P*	HR (95%CI)	*P*
Age^a^	0.998 (0.971–1.026)	0.884	1.005 (0.971–1.041)	0.772
Sex	0.796 (0.393–1.611)	0.526	1.233 (0.539–2.820)	0.620
pT^a^	0.432 (0.035–5.358)	0.513	0.447 (0.026–7.600)	0.578
pN^a^	0.769 (0.099–6.002)	0.803	0.579 (0.035–9.643)	0.703
pM^a^	NA	NA	1.467 (0.388–5.556)	0.572
Fuhrman Grade^a^	5.097 (2.091–12.426)	**0.000**	3.796 (1.384–10.410)	**0.010**
AJCC Stage^a^	8.758 (0.685–111.898)	0.095	13.606 (0.723–256.073)	0.081
COL6A1	3.052 (1.500–6.210)	**0.002**	2.211 (1.360–8.060)	**0.008**

Abbreviations: 95% CI, 95% confidence interval; HR, hazards ratio; FUSCC, Fudan University Shanghai Cancer Center;

*Bold type indicates statistical significance.

a Categorical variables: male patients versus female patients; pathological lymph node positive or otherwise; M0 versus M1;Fuhrman grade >2 or otherwise. T > 2 or otherwise, AJCC > 2 or otherwise.

### IHC results was associated with RNA level of COL6A1

**Figure 3:**
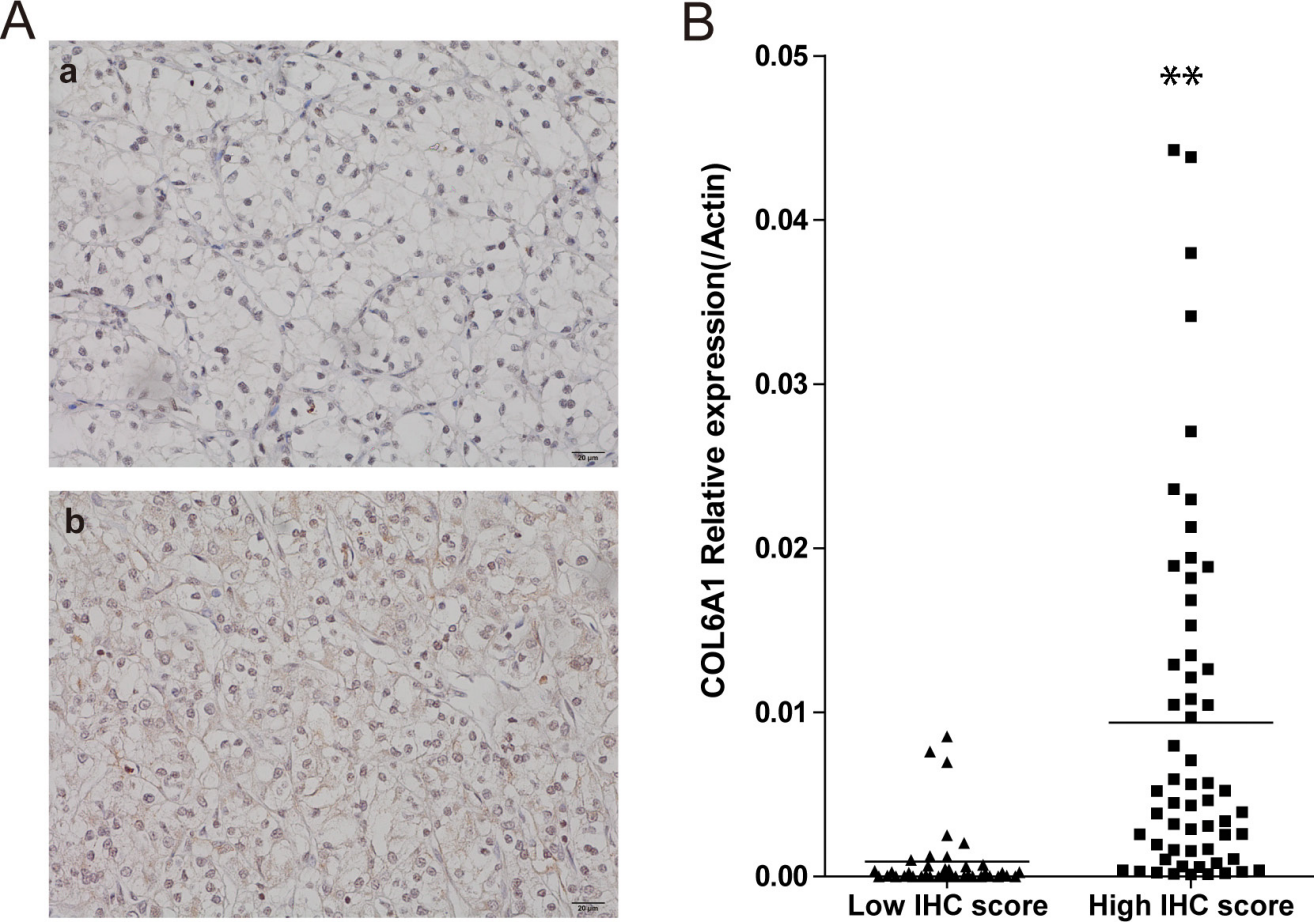
COL6A1 IHC score was correlated with mRNA level **A.** The two photographs (400X) showed negative (a) and positive (b) staining of COL6A1 in ccRCC. **B.** The comparison of relative COL6A1 mRNA level (normalized to β-actin) between low and high IHC score groups. Relative COL6A1 mRNA level in high IHC score group were significant higher than low IHC score group.

We then analyzed correlation of COL6A1 IHC results and relative mRNA expression in 103 patients who underwent surgery in 2009. COL6A1 was mainly expressed in tumor stroma of ccRCC (Figure 3A). On the basis of COL6A1 IHC score, 38.8% (40/103) Pca tissues showed low expression of COL6A1. The relative mRNA expression of COL6A1 was correlated with IHC score (*p* < 0.01, Figure 3B).

### Tumorigenesis of COL6A1

**Figure 4:**
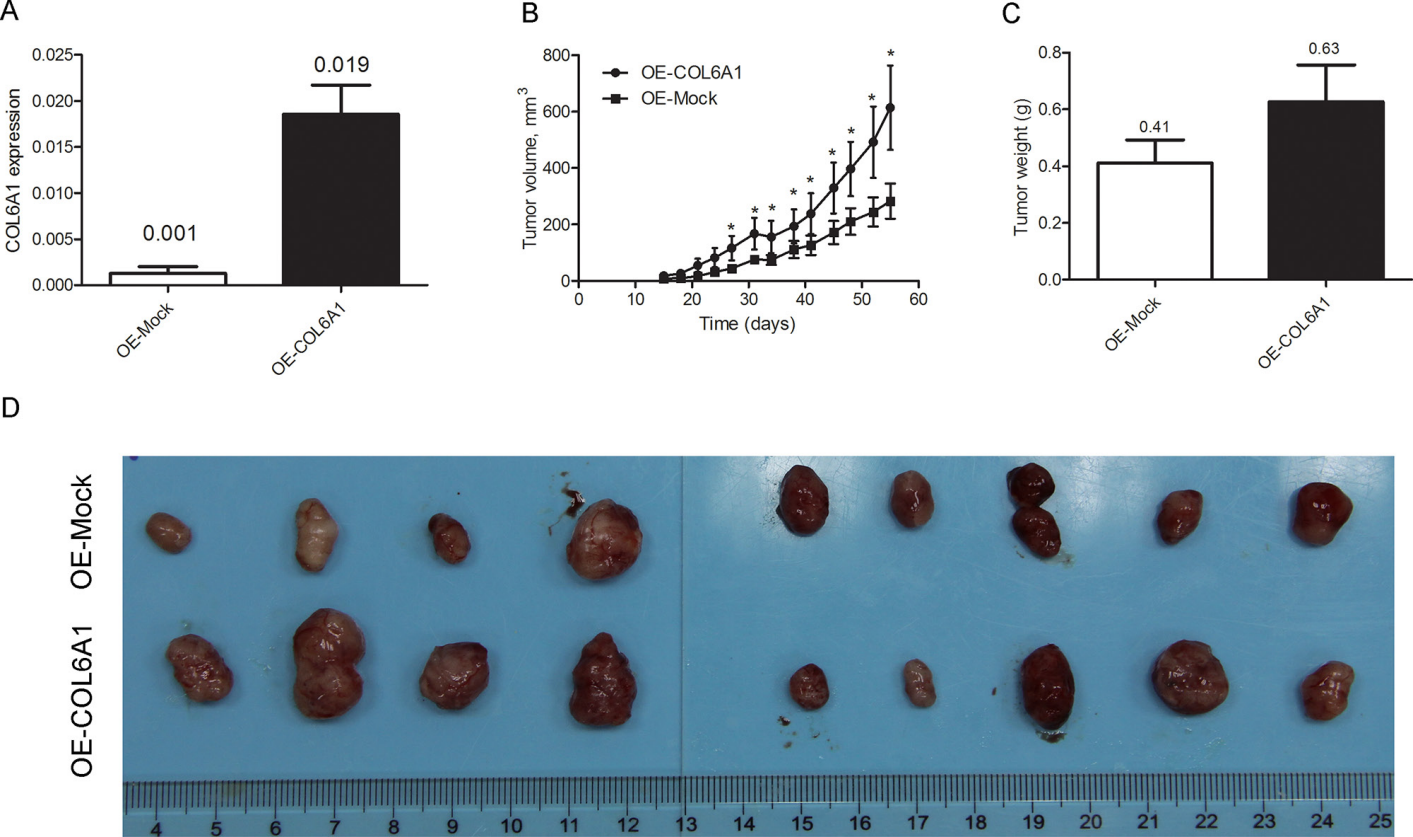
COL6A1 promotes 7860 tumor growth in nude mice **A.** 7860 cells were stably infected with the oe-COL6A1 and oe-Mock control particles. COL6A1 expression was determined by qRT-PCR and normalized to β-actin expression. **B.** COL6A1 promoted tumor growth in nude mice. Tumor volume was measured with Vernier calipers every 3 days and compared. **C, D.** The mice were humanely sacrificed on Day 56, and the tumors were weighed and photographed (*P* = 0.062). Data represent the mean ± standard deviation (SD).

To study the role of COL6A1 in tumorigenesis *in vivo*, we stably infected 7860 cells with lentiviral oe-COL6A1 particles to generate a COL6A1 overexpression cell line. After puromycin selection, 7860-oe-COL6A1 and 7860-oe-Mock cells were harvested for qRT-PCR analysis (Figure 4A). 7860-oe-COL6A1 and 7860-oe-Mock were bilaterally implanted into nine nude mice (5 × 10^6^ cells/site) and measured every 3 days. The mean tumor volume of oe-COL6A1 group was larger than that of the oe-Mock group from Day 27 (*P* < 0.05, Figure 4B). Mice were humanely sacrificed on Day 56. The mean tumor weight of 7860-oe-Mock control group was less than that of the 7860-oe-COL6A1 group (0.41 g vs. 0.63 g, *P* = 0.06), although this difference was not statistically significant (Figure 4C, 4D).

## DISCUSSION

In the present study, we found that the upregulation of COL6A1 expression was associated with poor prognosis of ccRCC patients in both TCGA and FUSCC cohorts. Moreover, the tumorigenesis study demonstrated that overexpression of COL6A1 promoted tumor growth *in vivo*. Taken together, our study indicates that COL6A1 represents a prognostic marker of ccRCC. To the best of our knowledge, the current retrospective study represents the first comprehensive survey of the clinical characteristics and outcome of ccRCC patients in relation to COL6A1 expression features.

The ECM provides essential signals to regulate cell growth and apoptosis. It can also influence responses to anticancer agents by regulating access to chemotherapy and potentially forming a physical barrier to promote resistance [9]. Collagens are one of the main ECM proteins, among which collagen VI plays an important role in cell signaling and cell migration [8, 10, 11] as well as interacting with a range of ECM components [12]. COL6A1, which is a conservative gene/protein in vertebrates and is present in all connective tissues [15], was recently found to be differentially expressed in astrocytomas [13]. Following global secretome analysis, Blanco et al. reported that COL6A1 is a novel mediator of bone metastasis [8, 14]. However its precise role in cancer is still poorly understood.

The mechanism by which COL6A1 expression is associated with a poor prognosis in ccRCC remains to be elucidated. A recent study on the von Hippel-Lindau tumor suppressor (VHL, also known as E3 ubiquitin protein ligase) suggested that VHL inactivation decreases H3K4Me3 levels through JARID1C, which suppresses tumor growth as well as COL6A1 expression [15]. In accordance with the results of our study, the restoration of COL6A1, which is expressed at low levels in the VHL−/− cell line 7860, promoted tumor growth. Thus, in VHL mutated patients, it can be speculated that COL6A1 upregulation indicates concurrence with inactivation of other tumor suppressors and may represent a signal of poor prognosis. Moreover, COL6A1 has been confirmed as a TGF-β/Smad target in human dermal fibroblasts [16]. TGF-β overactivation in cancer cells secreted and acted on surrounding stromal cells, these cells proliferate and increase TGF-β secretion. This over-abundance of TGF-β causes immunosuppression and angiogenesis and increases the invasive ability of cancer cells. Therefore, COL6A1 upregulation may be a consequence of TGF-β activation in tumors leading to poor patient prognosis. Recent studies have suggested that sorafenib inhibits TGF-β activity [17, 18]; thus, COL6A1 expression level may also reflect patient responses to sorafenib treatment in metastatic ccRCC although this hypothesis requires further investigation.

A major strength of the present study is that the samples were derived from two large populations with a long follow-up. However, prognosis of ccRCC could be influenced by many complicated factors such as staging, Fuhrman grade, surgical performance and response to adjuvant therapy; therefore, a single marker used in isolation will provide limited information. Another limitation of the current study is the expression of COL6A1 in the FUSCC cohort was determined by qRT-PCR analysis, which is not as specific as the RNAseq technique. In the subgroup analysis of PFS of sunitinib treatment, only 41 patients were enrolled, external validation is needed.

## MATERIALS AND METHODS

### Patients and samples

This study received Institutional Review Board approval from Fudan University Shanghai Cancer Center (FUSCC). Written informed consent was obtained from all subjects. ccRCC patients who underwent radical nephrectomy (RN) or nephron sparing nephrectomy (NSS) were retrospectively enrolled from 2009 to 2012. The clear cell subtypes were confirmed by experienced pathologists. Only patients with intact clinical data as well as frozen samples in the tissue bank of FUSCC were included. Demographic and clinical characteristics, such as age, sex, age at initial diagnosis, and stage at diagnosis (tumor, node, metastasis [TNM] classification) were obtained from electronic records. Frozen tissue samples obtained from these patients during surgery were stored at −70°C in the tissue bank of FUSCC. Patients were regularly followed up by telephone or in the clinic every 3 months until December 2014 and tumor recurrence, progression, metastasis, cause and date of death were recorded. A total of 41 patients received sunitinib treatment. Treatment and evaluation of efficacy were followed up by telephone or medical records.

COL6A1 expression and clinical data of the Cancer Genome Atlas (TCGA) database are available from the website of Cancer Genomics Browser of University of California Santa Cruz (UCSC) (https://genome-cancer.ucsc.edu/). In total, 497 primary ccRCC tumors from patients with detailed COL6A1 expression data were chosen from the updated TCGA database according to parameters defined in a previous study [19]. Patients without intact tumor expression data were excluded. The clinical data of these patients from TCGA cohort are shown in Table 1.

### Expression analysis

Total RNA was isolated from 288 ccRCC samples using TRIzol^®^ reagent (15596-026, Invitrogen). First strand complementary DNA was synthesized using a PrimeScript RT reagent kit (K1622, Thermo Scientific) for use as the template in SYBR Green real-time PCR assays performed using an ABI 7900HT (Applied Biosystems, USA). The expression level of mRNA was normalized to the level of β-actin [20]. The primers for qRT-PCR analysis of COL6A1 and β-actin were synthesized by Sangon (Shanghai, People’s Republic of China) using the following sequences:

COL6A1: F:5′-TCAAGAGCCTGCAGTGGATG-3′, R:5′-TGGACACTTCTTGTCTATGCAG-3′.

β-actin: F:5′-AGCGAGCATCCCCCAAAGTT-3′, R:5′-GGGCACGAAGGCTCATCATT-3′.

### Immunohistochemistry (IHC) analysis of COL6A1 in FUSCC cohort

All patients (*N* = 103) who underwent surgery in 2009 from FUSCC cohort were enrolled in the subgroup analysis of IHC. IHC were conducted as previously described method [21] with anti-COL6A1 (17023-1-AP, Proteintech, Chicago, IL, USA). All slides were reviewed by an experienced pathologist who was blind to this study to confirm diagnosis and IHC score. COL6A1 were mainly stained in tumor stroma. A staining index (range, 0–9) was calculated as a product of staining intensity (range, 0–3) and percentage of positive area (≤1%, 0; 1%–25%, 1; 25%–50%, >50%, 3). COL6A1 were divided into low (index 0 to 4) and high (5–9) expression groups. The correlation between protein level and RNA expression of COL6A1 in ccRCC patients were analyzed by *t* test.

### Overexpression particle construction

To construct the oe-COL6A1 vector, the COL6A1 gene coding sequence (CDS) followed by a 3′-FLAG tag sequence was inserted into the pCDH-CMV-MCS-EF1-Puro (CD510B-1) vector. Lentiviral particles were produced in HEK293T cells by co-transfection of oe-COL6A1 or mock vectors with psPAX2 and pMD2.G packaging vectors. Stable 7860 cell lines overexpressing COL6A1 or mock were obtained by lentiviral infection followed by puromycin selection [22, 23].

### Tumorigenesis study

The research was conducted in accordance with the Declaration of Helsinki and with the Guide for Care and Use of Laboratory Animals as adopted and promulgated by the United National Institutes of Health. All experimental protocols were approved by the Review Committee for the Use of Human or Animal Subjects of Fudan University. To determine the effect of COL6A1 on tumorigenesis of COL6A1, after confirming COL6A1 overexpression, 7860-oe-Mock and 7860-oe-COL6A1 cells were collected and injected subcutaneously into the right and left flanks (5 × 10^6^ cells/site) of nude mice (9 mice/group). To monitor the tumor growth, the volume was measured by using Vernier calipers on Days 3, 6, 10, 14, 17, 21 and 24 and calculated according to the following formula: volume = W^2^ × *L* × 0.5 (where W and L represent the largest and second largest tumor diameters [cm]) and then plotted. Mice were humanely sacrificed on Day 27, and the tumors were weighed and photographed.

### Statistical analysis

All statistical analysis was performed using SPSS software (version 17.0, IBM Corp., Armonk, NY, USA). Independent *t*-tests (for continuous variables) and Pearson’s *χ*^2^ tests (for categorical variables) were used. A two-sided *P*-value < 0.05 was considered to indicate statistical significance. The cut-point of COL6A1 expression was defined as the median. The overall survival (OS) was defined as the time from surgery to death due to any cause. The disease-free survival (DFS) was defined as the time of surgery to tumor recurrence, progression or metastasis in localized ccRCC. Progression-free survival defined as the length of time from the start of treatment to disease progression or death. Patients without events or death were recorded as censored at the time of last follow-up. Survival curves were constructed using the Kaplan–Meier method, with log-rank tests used to assess the differences between the groups.
